# Mental health and professional identity formation amongst medical students in Singapore: a qualitative study

**DOI:** 10.1186/s12909-025-08385-z

**Published:** 2025-12-10

**Authors:** Darius Wei Jun Wan, Nur Amira Binte Abdul Hamid, Lalit Kumar Radha Krishna

**Affiliations:** 1 Division of Internal Medicine, Singapore General Hospital, Outram Road, Singapore, 169608 Singapore; 2 Division of Cancer Education, National Cancer Centre Singapore, 30 Hospital Boulevard, Singapore, 168583 Singapore; 3 Division of Supportive and Palliative Care, National Cancer Centre Singapore, 30 Hospital Boulevard, Singapore, 168583 Singapore; 4 Duke-NUS Medical School, National University of Singapore, 8 College Road, Singapore, 169857 Singapore; 5 SingHealth Duke-NUS Medical Humanities Institute, 8 College Road, Singapore, 169857 Singapore; 6 Medical Affairs, National Cancer Centre Singapore, 30 Hospital Boulevard, Singapore, 168583 Singapore; 7 School of Health Sciences, University of Dundee, 11 Airlie Pl, Dundee, DD1 4HJ UK; 8 Palliative Care Institute Liverpool, Academic Palliative & End of Life Care Centre, Cancer Research Centre, University of Liverpool, 200 London Road, Liverpool, L3 9TA UK; 9 PalC, The Palliative Care Centre for Excellence in Research and Education, Dover Park Hospice, 10 Jalan Tan Tock Seng, Singapore, 308436 Singapore; 10 Health Data Science, University of Liverpool, Whelan Building, The Quadrangle, Brownlow Hill, Liverpool, L69 3GB UK

**Keywords:** Mental health, Medical students, Professional identity formation, Personhood, Stressors, Medical school, Anxiety, Asia, Singapore

## Abstract

**Background:**

Professional identity formation (PIF) is nurtured in medical school through the inculcation of the values, expectations and responsibilities of the profession—shaping belief systems that inform the professional identity and broader concepts of personhood. This developmental journey, however, places strain on mental health, with medical students facing higher rates of anxiety, depression and mental stress compared to non-medical peers. These experiences can impede effective PIF. Yet, studies that examine the intersection between PIF and mental health remain remiss, further compounded by the lack of research in localised, non-Western settings. To address these gaps, this study explores medical students’ lived experiences with mental health challenges in Singapore, focusing on their impact on PIF.

**Methods:**

Semi-structured interviews were conducted with 10 medical students on an oncology posting at the National Cancer Centre Singapore. Interviews were transcribed and analysed via deductive content analysis, guided by the Ring Theory of Personhood (RToP) that maps changes in belief systems across the Innate, Individual, Relational and Societal Rings.

**Results:**

Data analysis revealed three key domains: (1) stressors; (2) evolving self-concepts of personhood; and (3) adaptations. Medical students faced assessment-related, clinical, extracurricular and interpersonal stressors that introduced conflicts between emerging and existing belief systems. Personal adaptations to these conflicts were contingent upon the maturity of the internal compass and availability of personalised, enduring support systems. Students with a mature internal compass and rich peer, familial or collegial support engaged in healthy coping mechanisms that promoted adaptive growth in PIF. Conversely, an inexperienced internal compass and lack of support systems prompted maladaptive behaviour and placed further strain on the professional identity.

**Conclusion:**

Beyond short-term well-being interventions to mitigate mental health challenges, this study highlights the importance of nurturing the internal compass through longitudinal, individualised and context-sensitive support that sustains students through their professional development. Future work can explore how such approaches may be effectively operationalised and evaluated in different cultures and clinical contexts.

**Supplementary Information:**

The online version contains supplementary material available at 10.1186/s12909-025-08385-z.

## Introduction

Professional identity formation (PIF)—the process through which medical students learn to think, feel and act as physicians while adopting the profession’s values, beliefs, norms, expectations and responsibilities—is complex [1–5]. Shaped by sociocultural, professional, academic and moral values, beliefs, principles and obligations [3, 6, 7], PIF presents a dynamic developmental journey that involves the negotiation and integration of the personal and professional selves [8, 9]. This organic transition from “*doing the work of a physician*” to “*being a physician*” [10] marks a central tenet of medical education [8].

Medical schools have continued to finesse efforts to support this process [2, 11, 12], employing strategies such as mentorship programmes, where students learn, support and co-evolve through socialisation [4, 11, 13]; structured clinical exposure, with clearly defined roles, responsibilities and codes of practice that help students traverse from peripheral to central roles within the care team [14]; and reflective practice that promotes cognitive re-evaluation of identity-challenging events [15], such as complex patient encounters [16, 17] and morally ambiguous situations [18]. However, despite these interventions, barriers to effective PIF persist [3]. These include heavy academic demands and competing responsibilities [19–21], difficulty adapting to new clinical environments [22], and challenging peer or patient relationships [23]. Such stressors often intersect with emotional exhaustion, distress and anxiety, which can jeopardise students’ mental well-being and impede the healthy internalisation of professional values and responsibilities [24–28].

Recent studies reveal that medical students experience disproportionately high rates of anxiety, depression and mental stress compared to their non-medical peers [29–31], reflecting the cumulative pressures of clinical training and identity negotiation. Globally, the prevalence of depression and anxiety among medical students is estimated at 27% and 34% respectively [30–33], with suicidal ideation alarmingly reported in 11% of students across multiple countries [30, 33]. Such mental health challenges can result in adverse personal and professional repercussions, including burnout, heightened cynicism and social isolation [34–36]. Despite high prevalence of mental health challenges, most medical students refrain from seeking formal consultations or treatments [37], with fewer than one in six students with depression pursuing psychiatric help [33]. Some students show a preference for informal consultations while others resort to self-diagnosis or forgo help altogether [37–40]. Such evidence underlines the urgent need to redouble efforts in supporting students within the medical education landscape.

### Research gaps

While mental health and PIF have each been well-documented as critical concerns in medical education, studies that explicitly examine how mental health challenges interact with PIF remain remiss. Furthermore, the preponderance of studies grounded in Western settings may not adequately capture the unique sociocultural considerations in Asian societies [34, 41, 42], where notions on collectivism, stigma and emotional restraint can influence perspectives on mental health [43]. Similarly, failure to consider the social, cultural and contextual factors at play can limit the effective development of the professional identity [44]. These gaps thus call for a closer examination into the intersection between mental health and PIF in the Asian setting, particularly through qualitative exploration that offers nuanced, context-specific narratives underpinning lived experiences.

### Research aims and questions

This study aims to map the context-specific narratives of medical students’ lived experiences in multi-ethnic, multi-cultural and multi-religious Singapore. Semi-structured interviews are conducted with Year 3 and Year 4 medical students on a clinical oncology posting at the National Cancer Centre Singapore (NCCS), focusing on how mental health experiences influence PIF and personhood. Specifically, the study seeks to address the following research questions:

1. What mental health challenges do medical students encounter in medical school?
2. How do these challenges influence their professional identity and self-concepts of personhood?

We believe that insights from this cultural melting pot are useful in guiding loco-regional physicians, educators and medical schools in advancing more culturally attuned interventions and support systems.

## Methods

Grounded in an evidence-based approach, our semi-structured interviews were framed around concepts of personhood (‘what makes you, you’), drawing on the premise that clinical experiences can change belief systems and, in turn, reshape self-concepts of personhood [14]. These shifts in personhood often manifest as changes in self-identity, within which the professional identity forms an integral part. To capture these shifts, we employed the Ring Theory of Personhood (RToP), framed by Systematic Evidence-Based Approach (SEBA) to ensure transparency and reproducibility.

### Theoretical lens: the ring theory of personhood

**Fig. 1:**
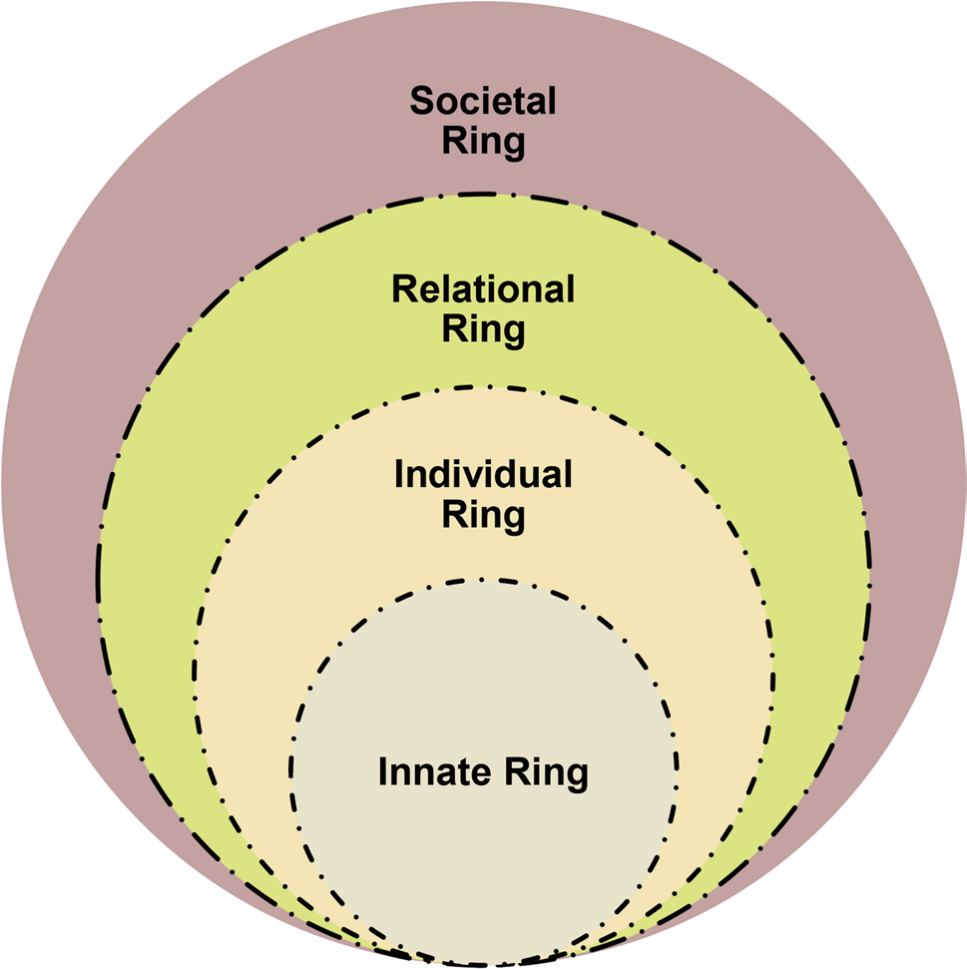
The Ring Theory of Personhood, adapted from Krishna and Alsuwaigh [45]

Advanced by Krishna and Alsuwaigh [45], the RToP is a conceptual model that charts changes in belief systems (Fig. 1). The authors posit that such belief systems—encompassing values, principles, norms and goals—reflect self-concepts of identity and, collectively, personhood [45]. New experiences, such as entry into medical school and complex patient encounters, may alter these belief systems, and by extension, reshape self-concepts of personhood [14, 46].

The RToP demarcates personhood into four concentric rings—the Innate, Individual, Relational and Societal Rings, each embedded with unique sets of belief systems [45, 47]. Here, the Innate Ring houses belief systems tied to the individual’s spiritual, religious, existential and demographic beliefs whilst the Individual Ring centres on belief systems surrounding personality, emotions and behaviour [48, 49]. Intimate relationships with family and friends guide belief systems in the Relational Ring while the belief systems in the Societal Ring are rooted in sociocultural, professional, legal and ethical norms, roles and responsibilities [24, 50]. It is proposed that exposure to mental health challenges in medical school can result in subconscious and conscious changes in these belief systems. These changes may take the form of *disharmony* when new beliefs, values and principles clash with existing ones within a single ring of personhood, or *dyssynchrony*, when new belief systems across two or more rings are in conflict [46, 48, 50, 51]. Here, the individual seeks to adjust regnant belief systems to accommodate insights, experiences, meaning-making and reflections. The intention behind such accommodations is to achieve *resonance*—a state wherein existing and new belief systems are in sync [47, 52, 53].

However, when *dyssynchrrony* and/or *disharmony* is left unaddressed and reinforced by new experiences or responses, it risks becoming embedded. These embedded belief systems then guide thinking, decisioning and conduct, forming what is referred to as the *internal compass* [12, 54, 55].

### Methodological framework: the systematic evidence-based approach (SEBA)

**Fig. 2:**
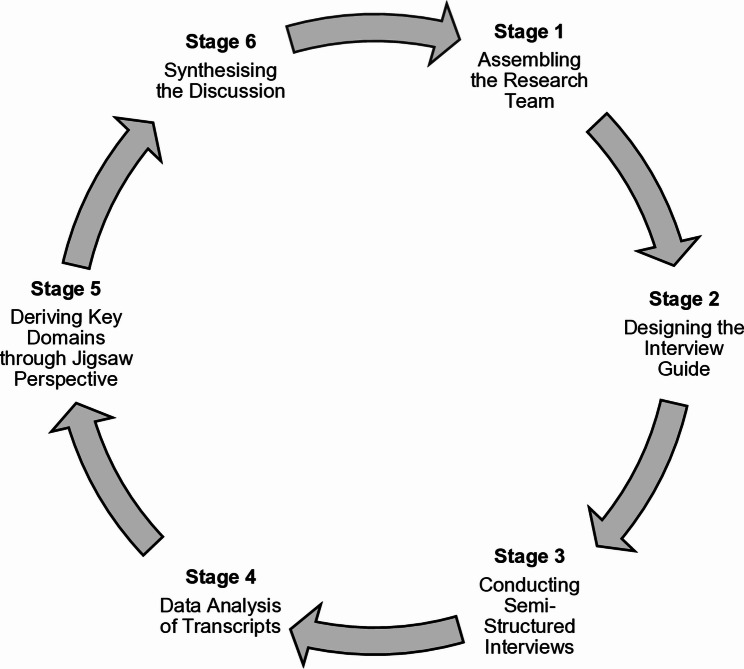
The systematic evidence-based approach, adapted from Tan et al. [54]

The SEBA methodology is a multi-staged, multi-analyst, structured approach to data analysis that safeguards methodological rigour, transparency and reproducibility [13, 56, 57]. To further strengthen these elements, this study adheres to the Consolidated Criteria for Reporting Qualitative Research (COREQ) checklist (see Additional File 1). Figure 2 depicts the stages of the SEBA methodology.

Ethics approval (CIRB reference number: 2023–2304) was obtained from the SingHealth Centralised Institutional Review Board. Written and verbal informed consent were obtained from all participants before the commencement of each interview.

#### Stage 1. assembling the research team

The SEBA methodology endorses a collaborative, multi-analyst approach to enrich data interpretation and reduce personal biases [57, 58]. This saw the active collaboration between a senior palliative care consultant as the principal investigator (PI), and a house officer and a research assistant as junior analysts, comprising the research team.

#### Stage 2. designing the interview guide

To build a comprehensive understanding of mental health challenges in medical school, the research team drew on a systematic approach to review prevailing literature in the field. This included articles on PIF, personhood and the cost of caring. Data from these reviews formed the basis of the semi-structured interview guide (Additional File 2).

#### Stage 3. conducting semi-structured interviews

Recruited through purposive convenience sampling, eligible participants comprised Year 3 and Year 4 medical students from the three medical schools in Singapore, who were posted to NCCS during their one-week clinical oncology posting.

An in-person presentation outlining the study objectives was conducted on the first day of each round of clinical posting. Attendants were invited to complete an anonymised online questionnaire, hosted on a secure government platform, in their own time. Aiming to assess the current mental health status of participating medical students, the questionnaire incorporated the Depression Anxiety Stress Scale 21 (DASS-21), designed to evaluate symptoms of depression, anxiety and stress using a 4-point Likert scale ranging from 0 (“did not apply to me at all) to 3 (“applied to me very much). This was further complemented by open-ended questions to explore self-concepts of mental health. Quantitative data from the questionnaire were not included in this qualitative paper and will instead form the basis of a subsequent study.

At its conclusion, participants could indicate their interest in a follow-up interview to explore their experiences in greater depth. Provided with contact details of the research team at the end of the questionnaire, interested participants reached out via e-mail, where they were provided with an information sheet and consent form. Upon receipt of signed consent forms, online semi-structured interviews were scheduled during or at the end of posting. The decision to conduct interviews virtually was based on logistical considerations to accommodate participants’ varying locations and availability. Participation was voluntary and participants were reimbursed with a $20-voucher for their time and contributions.

Of 18 students who completed the online questionnaire, 10 students participated in the follow-up interviews. Eight students did not opt for the interviews nor provided reasons for non-participation.

Between October 2023 to February 2025, interviews were conducted individually by three experienced female interviewers: a junior doctor with a medical degree, a researcher with a bachelor’s degree in psychology and a research assistant with a background in psychology. Their range of diverse yet related experiences facilitated empathetic questioning that elicited more detailed recounts from participants. This was further complemented by training in theoretical concepts of RToP, boosting recognition of subtle nuances and use of effective prompts for more robust interviews. Pilot interviews were conducted with the research team to refine the interview guide, thereby ensuring distinct alignment with the study aims. Minor revisions were made to improve the clarity of interview questions, flow and relevance.

While the first interviewer’s insider role as a junior doctor was advantageous in strengthening contextual familiarity, it also heightened the risk of social desirability bias that might result in alterations in participant responses due to perceived judgment. In mitigating these concerns, she was introduced solely as a researcher affiliated with the project. No personal or professional background information was provided that might have influenced participant responses. The remaining interviewers were external to the medical field and lacked proximity to the medical students, thereby minimising social desirability bias.

The interviews were conducted on an institutionally secured Zoom video-conferencing platform in vacant work offices, with no additional observers present. Conducted in English, each interview ranged from 30 min to one-hour and was audio-recorded with the participants’ consent. Field notes were not taken during the interview process.

Audio recordings were anonymised and transcribed verbatim for data analysis. Data collection and analysis occurred iteratively, with early analysis informing refinements to subsequent interviews, and data from new interviews shaping the ongoing analysis. Data collection concluded when two consecutive interviews with different participants yielded no new insights. Transcripts were not returned to participants. No repeat interviews were performed.

#### Stage 4. data analysis

Following the transcription of the audio recordings, interview transcripts were consolidated and underwent data analysis by the three research team members.

i. Content Analysis.

Data was analysed using Hsieh and Shannnon’s [59] approach to deductive content analysis, guided by a coding framework outlined by the RToP. The first junior analyst identified patterns in the transcripts that corresponded to elements of the RToP. Where data did not align with the pre-existing codes, new codes were assigned, allowing inductive insights external to the RToP to emerge organically and reducing the omission of novel insights. This iterative process was overseen and cross-checked with the second junior analyst and PI. Sandelowski and Barroso’s [60] approach to “negotiated consensual validation” was used to attain consensus among team members.

ii. Reflexivity.

Group and personal reflexivity were practiced to reduce biases and capture multi perspectives, ensuring a more robust analytical process.

As an educator and mentor, the PI’s proximity to a diverse spectrum of medical students strengthened contextual familiarity with participant experiences in medical school. This was complemented by his profound experiences as a senior palliative care consultant, providing deep insights on the complexities of clinical encounters faced by participants during their postings. His dual roles as educator and clinician thus enabled reflective consideration of his own assumptions during analysis.

The two junior analysts, each with distinctive backgrounds, further enriched the reflexive process. As a house officer, the first junior analyst was an asset in staying attuned to esoteric layers and contextual cues privy to his ‘insider’ role as a recent medical school graduate. This included familiarity with and appreciation of the dynamics, structure and demands of medical school. The second junior analyst is a research assistant, with a bachelor’s degree in sociology, experienced in publishing on PIF, mentoring and the cost of caring. Her background in the field boosted understanding of sociocultural factors and offered conceptual knowledge in analysing empirical findings.

Through iterative discussions and cross-checking of interpretations amongst the analysts, reflexivity was practiced both personally and collectively to ensure transparency and rigour.

#### Stage 5. jigsaw perspective

The Jigsaw Perspective builds on Moss and Haertel’s [61] posit that “a richer, more nuanced understanding of a given phenomenon” is attained when complementary qualitative data are reviewed together. It reimagines each finding as a jigsaw piece that, when combined with complementary pieces, portrays a more complete picture. The research team thus identified and combined complementary codes to form sub-themes, which were then further consolidated into key domains that framed the ensuing discussion [54, 63, 64].

## Results

10 medical students, four male and six female, on a clinical oncology posting at NCCS participated in the semi-structured interviews. Table 1 details the participant demographics.

**Table 1:** Participant demographics

Year of Medical School	Participant Number
Year 3	SN1-2, SN4-6
Year 4	SN3, SN7-10

**Fig. 3:**
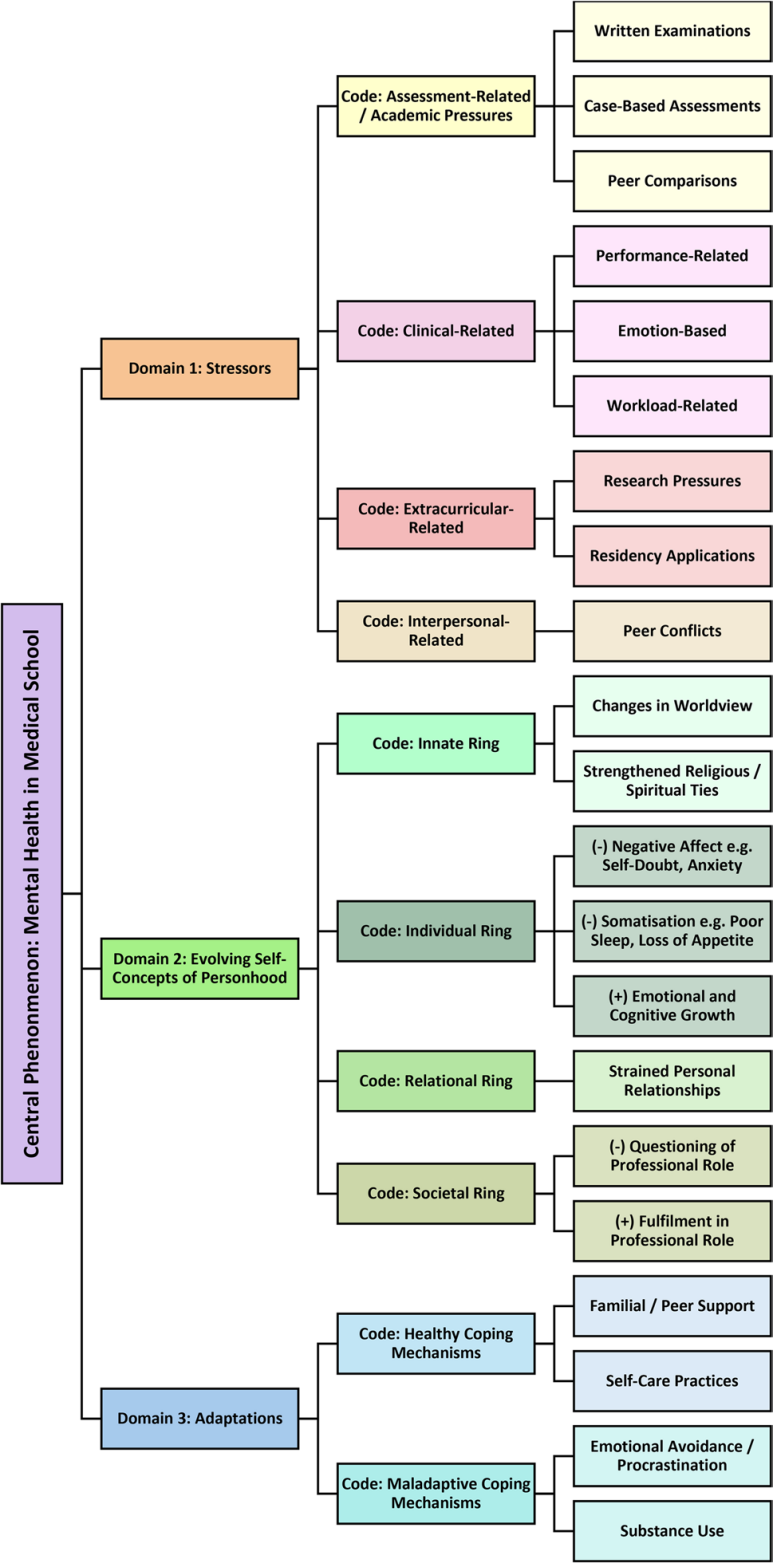
Coding tree

Three key domains emerged during data analysis: (1) stressors; (2) evolving self-concepts of personhood; and (3) adaptations. Figure 3 depicts the coding framework that guided this analysis. Participant feedback on the findings was not provided.

### Domain 1: stressors

Stressors refer to factors that negatively impact well-being. Stressors are highly personalised and context-sensitive, pivoting around four key areas: assessment-related, clinical, extracurricular and interpersonal.

#### Assessment-related stressors

Formal, informal, work-based and longitudinal assessments created a constant level of stress. At one extreme were written or end-of-posting examinations, which were perceived to carry significant academic or personal repercussions:

“I didn’t pass my promotional exams… I had to cancel my overseas community involvement programme that we had prepared half a year for and then go for the remedials… I think my mental health suffered then because it was the first time I failed at something that I really cared about.” (SN2).

The high stakes, unpredictable and broad nature of these clinical examinations further exacerbated stress and uncertainty:

“I really like how in [in junior college], the syllabus was very fixed… But for medicine, it’s really so broad… There’s really a wide range that you can prepare for… The biggest challenge for me is to train myself to be sharp enough to pick up all the signs and think on spot to formulate diagnosis.” (SN4).

These concerns mounted during case-based assessments:

“Another stress would be our consultants; they task us to get a history or examine this patient… The stress comes in… We’re not experts at doing it, so we might miss out something or we’ll do something wrong.” (SN1).

These high-stakes assessments exposed participants to a practice culture that amplified stress through peer comparisons and unspoken expectations (referred to as the hidden curriculum). Often self-deprecating and not necessarily situated in objective truths, this self-rated progress against peers and programme- and team-based expectations surfaced feelings of inadequacy and self-doubt that threatened mental stability:

“There were a lot of times where I felt inadequate because there are a lot of people around me who are doing far better. They always seem to be on the ball and then they didn’t seem burnt out…. There were times where I got burnt out and quite tired.” (SN9).

“You… compare… [with] your friends… and then you want to try and do better… I think all these kinds of things also adds up to the stress. And then especially in upper years, when our grades are not just pass/fail anymore, you get like alphabet, like A, B, Cs, there is more comparison.” (SN7).

This sense of inadequacy also extended to wider expectations and perceptions of success, creating internal tensions that deepened personal distress:

“I went to… a talk… about medical technology innovation. There were… overachieving doctors there who had sold businesses… During the talk, I was really inspired. But after talk… I felt really bad… These people did so many great things outside of medicine, but I can’t even do anything like that or be as successful during medical school. I feel like I’m just not as good enough.” (SN3).

“Every time they succeed, I’m really happy for them. But at the back of my head, there’s always this nagging feeling where I’m like…, ‘What are you doing compared to the rest of them?’” (SN10).

#### Clinical stressors

Transitioning from theoretical learning in the preclinical years to clinical rotations focused on real-life patient cases introduced new challenges that endangered mental health. Interactions with patients and senior consultants often highlighted perceived inexperience and failing expectations, creating performance-related stressors:

“At the start, where you don’t know what they would say, how they would react. Especially fresh into clinicals, like Year 3, when the doctor say they want the history from the patient…, you are like, ‘Oh, no. I never do this before. It’s my first time.’ I think it’s definitely stressful.” (SN7).

“As a medical student, we don’t want to say anything wrong to a patient… We need to be careful with what we say.” (SN1).

“Certain doctors or the nurses or assistants were a bit more harsh on us, which I would say did indeed contribute a bit to the stress of it all.” (SN9).

The nature of patient encounters posed an emotional stressor. For most participants, clinical postings in oncology presented their first exposure to intense suffering and death, often exceeding their capacity to process and manage such experiences. Emotional responses were amplified by perceived relational similarities, triggering distressing reflections on personal mortality or the potential loss of loved ones:

“When I realised that [the patient] had two sons… I was… thinking about how she’s kind of like my mom’s age and then I was… sad for her. And I was also thinking about how it could be easily at my own parents in that situation…. She started crying… So, I just started crying.” (SN3).

“Before, I was really, really afraid of death… I mean, I go into the A&E and one person’s alive and then a couple of minutes later, he’s already passed on.” (SN10).

Workload-related stressors added further pressure to clinical postings. Extended hours, frequently spanning morning to evening, coupled with ongoing academic responsibilities, severely diminished time for recuperation that jeopardised mental well-being:

“From eight to six is really a bit insane every day… There’s still time at night, but it’s really so tiring that I cannot study at night and there’s still so many textbooks [to read].” (SN4).

#### Extracurricular stressors

Within the competitive landscape of residency applications, participants described a strong pressure to pursue research and other scholarly endeavours to strengthen their prospects. The substantial time and effort required of such commitments—often undertaken voluntarily but perceived as necessary—added to their mounting workload and frequently encroached upon academic responsibilities. Collectively, these extracurricular stressors diminished opportunities for rest and self-care, contributing to heightened stress:

“The biggest stressor is really whether I am doing enough research… compared to them.” (SN10).

“A lot of people want to do research… I would say probably a voluntary stressor that they choose to pick up because they want to do research.” (SN7).

“Everyone’s always talking about what kind of specialty they want to get into and how competitive residency is… We are pretty stressed about whether we can get into the specialty that we want.” (SN8).

#### Interpersonal stressors

Interpersonal conflicts between peers in medical school presented an additional source of stress for participants. While clinical groups—small teams of six medical students working closely together during postings—offered community and support within a team-based learning environment, they were also sites where conflicts could arise. Disputes, gossip and exclusion left some participants feeling isolated, placing strain on their mental health:

“I find it difficult when there are people who gossip, I just find that bit two-faced. I feel quite hurt by that… [It] affected me personally… when there were people who talked behind my back… I felt a bit isolated in that sense.” (SN5).

“I think that some students, they are lonely, like they don’t have a lot of friends they can talk to… I think particularly for my batch, there’s a lot of problems with the clinical groups… like one person wouldn’t really fit [in] with everyone… and don’t really have anyone they can consult in.” (SN1).

### Domain 2: evolving self-concepts of personhood

Stressors influenced belief systems within the Innate, Individual, Relational and Societal Rings, reshaping self-concepts of personhood.

#### Innate ring

Distressing end-of-life encounters prompted re-evaluations of perspectives on life and suffering. This included a reframing of experiences—shifting from a myopic focus on immediate challenges to a broader appreciation of the continuity of human struggles. This wider frame enabled participants to address them more manageably, fostering balance amidst the dynamic nature of medical training:

“We’re so into the struggles we face. So, it helped me scale out… What is one little struggle or one little hurdle amidst everything?… Just zooming out and adopting a more open view about things and then realising, actually maybe I’m just caught in my emotions in the moment.” (SN10).

Others sought solace in their faith when faced with distressing circumstances, reaffirming their religious or spiritual ties:

“Stressors make you a bit more open-minded, make you consider religions a bit more, especially when you’re not religious. I know of some friends that have considered other religions because of these things.” (SN8).

#### Individual ring

Participants described a combination of negative affect and somatisation to persistent stressors. Engaging in complex situations often precipitated feelings of inadequacy and anxiety that perpetuated a negative self-image and threatened self-confidence:

“Feelings of inadequacy and always the fear of failure, feeling like I wasn’t good enough to be in medical school… I was like, did I really enjoy doing medicine? Am I really going to do this for the next four or five years and maybe for life as well?” (SN9).

“Because I thought I was anxious, I put a lot of labels on myself where I was like, ‘Oh, you won’t be able to handle high-stress situations. You won’t be able to do specialties that require a lot of stressors.’ And then I shut myself down on a lot of things.” (SN10).

“I was overwhelmed by a lot of emotions, thinking that… I was a failure, that I was not cut out to do medicine… Everyone in the batch would therefore think that I’m not capable… I felt like unworthy of my spot in medicine… of becoming a doctor.” (SN2).

These experiences significantly compromised emotional health as some participants experienced waning motivation, mood swings and in extreme cases, suicidal ideation:

“I’d lose a bit of motivation to study… That would just naturally impact your mood and the way you take care of yourself as well. You just don’t feel very good about yourself… It will impact your studies.” (SN9).

“There was this period where I was super-duper, duper stressed… And then was just very, very, very low mood. Very, very anxious also.” (SN10).

“I don’t really consider suicide seriously… but this thought does come across my mind, like, if only if all these can end and all the suffering in the future years will not happen.” (SN4).

These emotional and psychological challenges placed burden on physical health. Participants reported poor sleeping habits, declining appetite and weight fluctuations:

“For a couple of months, I just had no appetite, I couldn’t sleep.” (SN10).

“I’m really stressed and I actually don’t cope that much. I sacrifice a lot of my sleeping, a lot of my eating.” (SN6).

Yet, confronting distressing situations also inspired growth, serving as catalysts for emotional and cognitive development. Some participants described shifts in perspectives, including greater acceptance of individual timelines, a redefinition of success and an emphasis on living in the present:

“I realise that everyone has their different paths that they take and everyone’s running towards the finish line. Just that there are many ways to get to the finish line. And them going through a few other things, or going a few steps ahead of me, doesn’t take away the fact that I will still reach the finish line one day.” (SN10).

“It makes me think, like, okay, there’s more to life than just, being competitive and trying to win awards and stuff.” (SN3).

“I think of the future a lot. I was always telling myself that I’m going to be happy in the future… I wasn’t happy with me now in my daily life. And I think that was an issue that I had to address, like I have to just be happy with where I am every day. I shouldn’t be waiting for that future.” (SN5).

#### Relational ring

The demands of medical training often infringed upon participants’ relational ties, leaving minimal time for family and friends and placing strain on these relationships:

“My mom has suffered the most because every time there’s exams, she will say she’s very afraid to talk to me because she didn’t know what she say can make me even more upset…That sort of strained a bit my relationship with my mom. But after the exam, I would just apologise to her… tell her that I was very stressed.” (SN4).

“Doctors don’t have a lot of time for their own family… I would say that it’s difficult because I’m someone who is more family-oriented.” (SN5).

#### Societal ring

Self-doubt and uncertainty surrounding career prospects festered with prolonged exposure to such stressors. This saw the erosion of professional confidence as some participants questioned their suitability for medicine and future roles as physicians:

“I was also doubting whether being a doctor will be satisfying for me. I realised that I didn’t really enjoy the environment of practicing medicine in the hospital.” (SN2).

For others, deeper immersion in patient-centered care led to a sense of fulfilment in their professional roles as medical students and future physicians:

“I feel very fulfilled as a medical student. Times where I feel like I should have picked another discipline…, I get reminded about how this discipline [is] a very unique one… Patients just tell their entire life story without holding back… There is this large amount of trust… and it’s something I do not take for granted.” (SN10).

“I feel a bit more fulfilled… in our clinical years… There is more involvement on our part and I feel that does help a bit to keep me going because Year 1 and Year 2 is really just studying. You don’t really feel involved in the team or the care of the patient and I guess that affects you. It doesn’t motivate you as much as compared to when in clinical years.” (SN9).

### Domain 3: adaptations

Participants described a range of healthy and maladaptive adaptations in managing the stressors faced.

#### Healthy coping strategies

Participants often turned to familial and peer support as a buffer against stressors. Shared struggles amongst peers fostered comfort and reassurance within a community whilst externalising complex feelings with family members provided an avenue for processing and relief:

“I realised it seemed healthier to share and realise that other people are also experiencing it together. We were able to better support each other through the similar shared experiences. To me, that was a better way of coping compared to previously.” (SN9).

“I’ll talk to either [my boyfriend] or my mum… They will help me get through it. Or… I do things to distract myself, like different activities… with people.” (SN3).

Additionally, participants engaged in self-care practices, such as exercise, journalling and meditation, which offered a respite from the rigorous demands of medical school. These activities facilitated self-reflection and re-evaluation of perspectives that promoted balance and mental well-being:

“Journaling and exercise… When I journal, it allows me to process it better because I can… observe my emotions playing out… I recognise the pattern of thought or emotions that I’m falling into… It helps me to process.” (SN2).

“A lot of talking to friends, jotting things down, meditation…. Hearing different people’s perspectives about things helps me to…. understand what [I’m] truly worried about.” (SN10).

#### Maladaptive coping strategies

For others, fear of confronting these challenges led to emotional avoidance and disengagement. These maladaptive behaviours lead to substance use, procrastination and an unhealthy preoccupation with work. While such practices offered temporary relief, the underlying issues remained, impairing emotional regulation in the long run:

“I have a bad habit of ignoring, avoiding these external problems if I become more busy with medical school.” (SN1).

“I was surprised to find that some medical students do actually smoke, but just not in front of others… Some people do… drink alcohol and not recreationally.” (SN6).

“Most of the time, I just wait… until this the main source of my stress is over. Because for me, the main stress is academic, so I’ll just wait until the exam is over… I don’t think I have found a very good way to really, really remove the stress.” (SN4).

## Discussion

This study explores Singaporean medical students’ experiences with mental health challenges and their influence on PIF and personhood. Captured by the RToP, the findings highlight how assessment-related, clinical, extracurricular and interpersonal stressors can shape students’ belief systems across the Innate, Individual, Relational and Societal Rings and, in turn, mould their evolving sense of self and their developing professional identity.

Mirroring prevailing literature [28, 62–66], assessments emerge as powerful stressors in medical school. The high-stakes nature of written examinations, case-based assessments and supervisor evaluations frequently trigger feelings of inadequacy and self-doubt. This appraisal-driven anxiety is further shaped by the hidden curriculum in medical education—described as underlying influences and dynamics external to the purview of the formal curriculum—where competence is often implicitly equated with self-worth and institutional value [67]. In Singapore’s highly competitive landscape, this pressure is further compounded by extracurricular stressors, such as research outputs, leadership roles and residency placements, commonly rationalised as professional preparation amidst the local *kiasu* (local colloquial term for fear of losing out) mentality [68, 69]. This achievement-centered culture that endorses continual self-optimisation over wellness [70] is particularly pronounced within Singapore’s high-performance and collectivist educational culture [68, 69, 71]—amplified by social comparisons that implicitly purport peer achievements as benchmarks of success, as our findings suggest. Such dynamics reinforce the relational dimension of identity formation, where belonging to the learning community seemingly rests upon performance. Falling short of these expectations predisposes to what SN2 describes as feeling “unworthy of [their] spot in medicine.”

Clinical stressors further add to this stress. The transition from classroom to clinic is a critical but vulnerable phase in PIF [72] as students bridge the gap between theoretical learning as laypersons and direct patient care as professionals, laden with new challenges and emotional demands. Aligned with existing research [24, 73–77], our findings show that distressing patient interactions evoke strong emotional and moral distress. Here, the oncology posting marks many medical students’ first exposure to death and suffering, eliciting profound empathy, grief and fear of mortality, triggering shifts within the Innate Ring.

Collectively, these stressors not only threaten students’ mental health but also destabilise their evolving sense of professional identity. This often manifests as *dyssynchrony* between professional expectations and performance in the Societal Ring, and self-deprecation and doubt in the Individual Ring. Tinged with cynicism, participants’ initial idealistic views that frame medicine as a calling or noble pursuit shift to more pragmatic perspectives. Compromised personal ties, poor work-life balance, the mental toll of anxiety and depression, as well as the ‘fight or flight’ responses to stressful encounters, further reiterate the realism of the role. These findings echo that of Silveira et al.’s [78] study that explicates the state of dissonance arising from the disconnect between “what one desires or idealises, and what one observes or practices”. The implications for such dissonance in one’s professional identity are significant, culminating in depersonalisation, burnout, waning perception of quality of life, diminished empathy, depressive disorder and suicidal ideation [78–82].

Yet, our findings also reveal that mental health challenges can catalyse growth and foster PIF. Working alongside healthcare providers in difficult circumstances, the camaraderie forged in these stressful environments, accepting their fallibility with humility and the sense-making of challenging encounters sustain continued engagement and motivate many. For some, these insights encourage engagement with spiritual support and holistic evaluations of their experiences. Such a perspective helps medical students navigate inevitable struggles and failures in medical school. This more balanced sense of self inspires a move from external validation to more intrinsic motivation—reshaping belief systems in the Innate and Individual Rings and, in turn, fostering maturation within the Societal Ring. This evolution is marked by growing awareness of the impact of societal expectations on clinical and professional practice. Primarily, the ability to step back and situate challenges within a wider backdrop improves overall decision-making and coping mechanisms, particularly amidst a rise in cynicism in the clinical years.

### Internal compass

We posit that this ability to retreat, reframe and grow from adversity is contingent upon a maturing *internal compass*—one’s internalised roadmap of moral, ethical and professional values that guides decision-making and shapes meaning-making of experiences [12, 54, 55]. We extend this line of thinking and advance the notion of the *internal compass* as a self-regulatory mechanism that enables students to appraise, interpret and recalibrate dissonant experiences arising from mental health challenges to achieve *resonance* between new and existing belief systems. When activated by stressors, this adaptive feedback process allows personal and professional identity to remain coherent amidst conflicting contextual demands that precipitate *dyssychrony* and *disharmony*.

We suggest that the outcome of this process—adaptive growth or maladaptive strain—is dependent on the maturity of the *internal compass* and the availability of enduring, personalised support systems. Students who receive effective peer, familial or collegial support are more equipped to engage in healthy adaptations, such as adopting intrinsic measures of success and practicing self-reflection that inspire the reframing of dissonant experiences as opportunities for recalibration and growth. Without adequate support, however, the *internal compass* may advocate for maladaptive practices, including emotional avoidance and perfectionism, simply to gain *resonance* with the challenges faced.

**Fig. 4:**
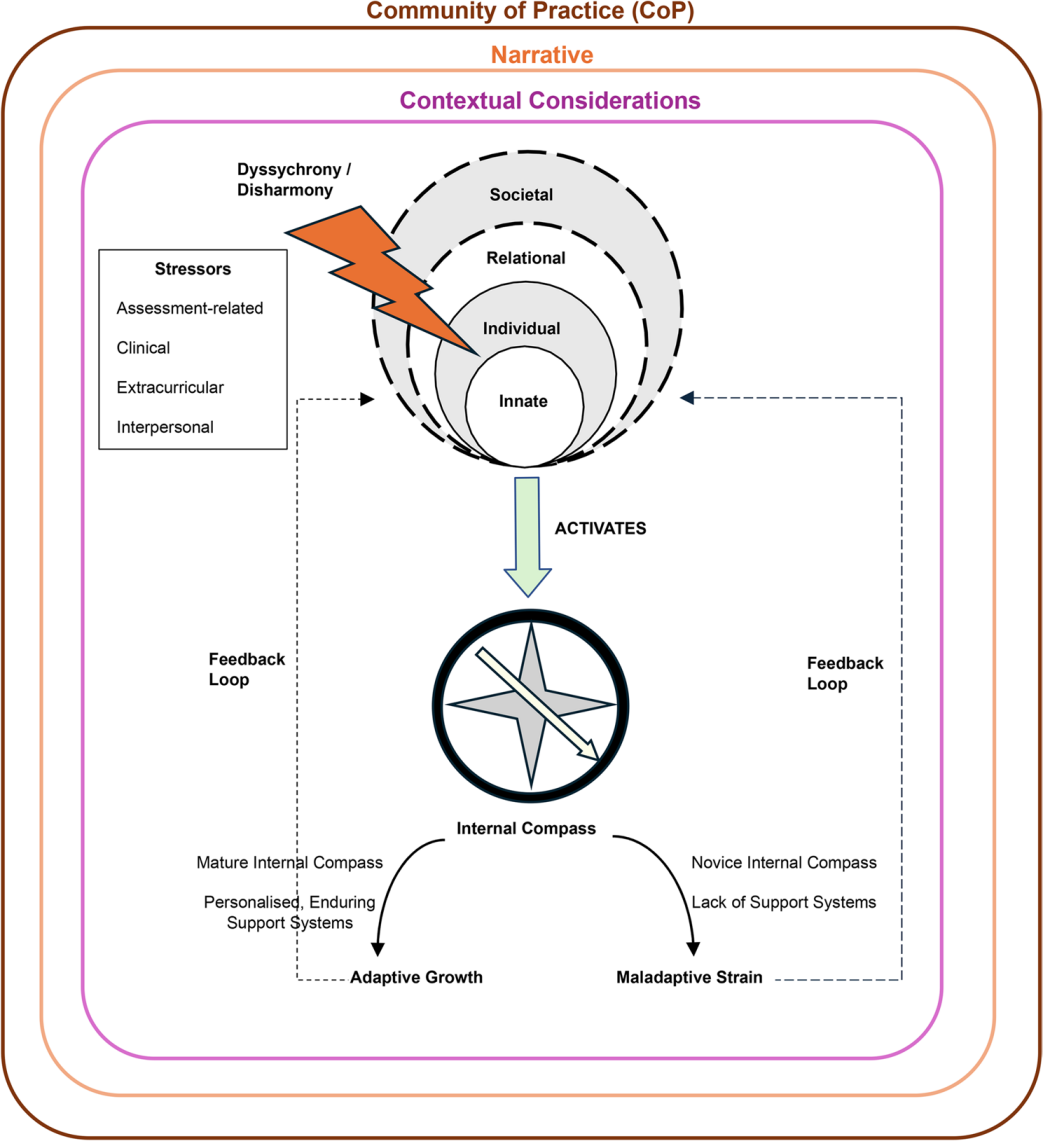
Self-regulatory mechanism to mental health stressors. Stressors introduce conflicts between existing and emerging belief systems within (*disharmony*) or between (*dyssychrony*) rings of personhood, activating the *internal compass*. A mature *internal compass*, coupled with personalised and enduring support systems from peers, family or colleagues promotes adaptive growth through the adoption of healthy coping strategies. This introduces a feedback loop to mediate the disparity between emerging and existing belief systems, facilitating the alignment of values to achieve *resonance* that strengthens the professional identity. Conversely, an inexperienced internal compass and lack of support systems prompt maladaptive behaviour, introducing a negative feedback loop that places further strain on the professional identity

Taken together, our study forwards a novel exploration of the intersection between mental health and PIF from a RToP-based lens and how the *internal compass* functions as a self-regulator to mediate adaptive or maladaptive responses that inform the professional identity. We integrate these findings into a succinct conceptual model that details this regulatory response to mental health stressors faced by medical students (Fig. 4).

### Implications for medical education

The centrality of the *internal compass* in governing the responses to mental health challenges and the subsequent ramifications on PIF underscores the need to move beyond short-term stress management and well-being interventions [83] to more longitudinal strategies that cultivate the *internal compass*.

This closer focus on refining the *internal compass* can be embedded within training programmes that function as a community of practice (CoP) [11, 12, 84, 85]. Such communities aim to foster the mutual development of individuals with shared competencies, resources, values and capacities as they collaborate towards common goals defined by the programme’s codes of practice, expectations, roles and responsibilities [12, 86]. These CoP-like programmes support students’ progressive transition from peripheral participation to more central roles [11, 12] whilst shepherding the socialisation of shared beliefs and inculcation of a common programme identity.

Our finding suggests that this immersive learning process brings about highly personalised experiences unique to each medical student. Trained faculty within such programmes—through advising, tutoring, role modelling, networking, coaching, supervising, guided reflections and mentoring (forming the ‘mentoring umbrella’) [12, 46, 87]—can provide the continuity and personalisation needed for students to make sense of their experiences and develop their *internal compass*. This mentoring umbrella accounts for each student’s narrative, context, competencies, resilience, support systems and engagement with the learning process to shape individualised meaning-making, coping mechanisms and unique adaptations that are congruent with both the student’s evolving identity and the broader values of the programme. Such support is especially needed at transition and pain points when stressors begin to overwhelm coping abilities and mental well-being.

We suggest that nurturing the *internal compass* in this manner helps embed belief systems that best reflect PIF and address *dyssynchrony* and/or *disharmony* in ways that remain concordant with the students’ evolving professional identities. Longitudinal strategies—such as e-portfolios; personalised, prompt and appropriate feedback; guided reflections; timely and accessible advice; and holistic mentorship, particularly through RToP-based assessments and individualised guidance—may thus offer the most promising means of supporting PIF while safeguarding mental well-being.

### Limitations

This study is not without limitations. First, the reliance on single point semi-structured interviews introduces the possibility of recall bias. Second, the conduct of all interviews by female interviewers runs the risk of gender-related bias. Given that gender-matched interviews are more likely to strengthen rapport, comfort and disclosure of personal experiences [88], male interviewees may have withheld or moderated their responses, potentially influencing the depth and nature of the data collected. Third, the absence of field notes during interviews may have further limited the nuance and richness in data analysis, following the omission of plausible non-verbal cues uncaptured by interview transcripts. Fourth, the unique contextual factors of oncology—such as frequent exposure to death and the emotional intensity of managing suffering—may limit the transferability of these findings to other specialties with higher recovery rates or less sustained contact with terminal illness.

## Conclusion

Medical school marks the transformative period in which PIF begins to take root as students internalise the values, expectations, norms and roles of the profession. However, in Singapore’s high-performance, competitive and collectivist educational culture, assessment-related, clinical, extracurricular and interpersonal stressors place medical students at heightened risk of mental health challenges. These experiences can challenge existing belief systems and hinder effective PIF. A mature *internal compass* is central to mediating such conflicts to foster adaptive growth. Rather than appointing isolated interventions, our study highlights the importance of nurturing this *internal compass* through longitudinal, individualised and context-sensitive support that sustains students through their professional development. Future work can explore how such approaches may be effectively operationalised and evaluated across different cultures and clinical contexts.

## Supplementary Information

Supplementary Material 1: Additional File 1. COREQ Checklist.

Supplementary Material 2: Additional File 2. Interview Guide.

## Data Availability

All data generated or analysed during this study are included in this published article and its supplementary information files.

## Abbreviations

PIF: Professional Identity Formation
NCCS: National Cancer Centre Singapore
RToP: Ring Theory of Personhood
SEBA: Systematic Evidence-Based Approach
COREQ: Consolidated Checklist for Reporting Qualitative Research
PI: Principal Investigator
DASS-21: Depression Anxiety Stress Scale 21
CoP: Community of Practice
