# Supplementary material for: Mental health and professional identity formation amongst medical students in Singapore: a qualitative study
Source: BMC Med Educ. 2025 Dec 10;26:68. doi: 10.1186/s12909-025-08385-z (PMC12801576; doi:10.1186/s12909-025-08385-z)
Supplement: Supplementary file 2 — Supplementary Material 2: Additional File 2. Interview Guide. [file 12909_2025_8385_MOESM2_ESM.docx]

**Additional File 2. Interview Guide**

| **Introduction** | - Hi <NAME>, thank you very much for agreeing to this interview.   I am <NAME> from the Division of Cancer Education and I will be interviewing you today. The interview will take approximately 45 to 60 minutes. But if you have to leave earlier, please let me know.   The focus of this interview is to better understand how your personal experiences in your medical training journey has grown your professional identity and shaped you into the person you are, or “your personhood”. We believe that “personhood” is determined by multiple domains which I will cover later.  Another thing to note is that this interview will be audio recorded and anonymised for analysis. The audio recording will be deleted after the study is completed. If that’s ok, may I have your permission to record and begin the interview?  Please note at that any point the interview can be stopped if you wish. In addition if there are questions that you would rather not answer or may stir up distressing memories or thoughts please let me know and we can talk about another topic. |
| --- | --- |
| **Topic Areas** | Questions |
| **Introduction, context, and Experience Questions** | 1. Firstly, could you share why you chose to enter the field of medicine?   Prompt if needed;   - What experiences inspired you? What values and beliefs guided you, and what motivations and goals were behind your decision?   [Response]   1. Could you tell me a bit about yourself your stage of training and a bit about your desired specialty? How has your training shaped the path towards making healthcare your career?   [Response]  Thank you for sharing. I would like to ask you about your sense of professional identity. When thinking of professional identity, you might want to think of the qualities, values, or roles that feel central to you as a physician-in-training. The formation of professional identity at this stage for you might refer to the process of learning and internalising the traits of your version of an ideal physician – and I would like to unpack the learning experiences that have contributed to this for you.   1. Given this definition, how would you describe your professional identity at this stage in your training?   [Response]   1. Looking back, has your understanding of what it means to be a physician changed over the course of your training?   [Response] |
| **Emotionally challenging LEARNING case**  **Emotionally challenging CARING case**  **Any other experiences**  **Comparison of impact on well-being (burnout, personal life, relationships)**  **Comparing the stressors in terms of type of emotional strain** | Thank you for sharing. I'd like to discuss your experiences facing emotionally challenging situations throughout your training. The concepts of “cost of learning” and “costs of caring” will be brought up, and it refers to the impact that difficult learning and caring experiences have had on you. When we think of “costs”, I would like you to think about learning and caring experiences that have contributed to feelings of burnout, compassion fatigue (whether from patients or from empathising with other staff), or secondary traumatic stress from witnessing the suffering or deaths of patients. We may also think of moral distress, which is a form of distress brought about by a feeling of conflict between your personal beliefs and what is expected of you professionally or societally.   1. Can you describe an experience during your training that was especially emotionally challenging for you? For example, a challenging class interaction or assessment, or ward teaching?   Prompts:   - What made this situation so challenging for you? - How did you feel during and after the experience? (e.g., stress, anxiety, sadness, fatigue, etc.)   [Response]   1. Can you describe a CARING experience during your training that was especially emotionally challenging for you? For example, a challenging patient or complex case? Just for some context, the reason why I’ve asked for two examples is to see whether there is a distinction between the challenges of learning and the challenges of caring for you, as a student, who has both types of experiences.   Prompts:   - What made this situation so challenging for you? - How did you feel during and after the experience? (e.g., stress, anxiety, sadness, fatigue, etc.)   [Response]   1. During your training so far, have there been any other memorable instances where you felt you were encountering feelings of burnout, compassion fatigue, or moral distress? How did you recognise that you were in a state of burnout or distress?   Clarification if needed:   - Burnout: Emotional exhaustion, detachment - Compassion fatigue: Running out of empathy - Moral distress: Feeling like you know the “right” thing to do, but you are unable to   Prompts if needed:   - Have you been able to notice what types of situations tend to trigger such feelings?   [Response]   1. What impact did these challenging learning experiences and feelings of burnout/distress have on your personal well-being, and were the emotional impacts of each case different? Did they affect your life outside of schooling? E.g., relationships with family?   Further prompts to tease out differences:   - How distinct were the emotional IMPACTS (significance) of each experience? (will be explored more in PIF section) - Was one worse (severity) than the other?   [Response]  Summarise the differences noted in interviewee’s COL and COC experiences. For EXAMPLE:  It sounds like the main emotional burden and strain in your learning challenges [COL] mainly revolved around the stress of managing content demands and perhaps feelings of self-inadequacy. Whereas for the challenging caring experience [COC] you faced, the main burden sounds like it’s coming from a kind of empathic strain – feeling overstretched from a time where you had to confront patient suffering and empathise with the patient.  Would you say that this is accurate?   1. Could you elaborate more on the feelings of empathic strain led on by your challenging caring case? In particular, I’d like to know more about how this empathic strain might have further amplified the costs of caring for you/ might have led to further feelings of burnout, compassion fatigue, and secondary traumatic stress.   [Response]   1. Do you feel that there have been long-term, cumulative effects of empathic strain from experiencing numerous challenging caring cases similar to the one you have described? What have these long-term effects been?   Prompts/ examples:   - Impacting relationships with patients - Loss of empathy (could be healthy or unhealthy) - Chronic sense of detachment   [Response] |
| **Immediate Response and Coping; Reflection on Coping Evolution** | 1. How did you feel during and after the experience and how did they impact your personal wellbeing? (e.g., stress, anxiety, sadness, fatigue, etc.) Were the emotional impacts of each case different?   [Response]   1. How did you cope with those emotions in the moment? For example, did you try to talk to someone, distract yourself, reflect on it alone, etc.? Were there any similarities or differences between your immediate handling of the two situations?   [Response]   1. Would you say that the culture of the school of medicine has had a role in shaping how you manage such challenges? For example, is there a big emphasis on talking out your challenges – or conversely is there perhaps a pressure to “toughen it out”?   Note: to just take note of the points raised regarding organisational culture (e.g., approaches to challenges, attitudes towards help-seeking etc.) and to further explore these points in the last section.  [Response]   1. Did you take away any memorable lessons or meaning from these incidents? For example, did it change how you view your role as a student/ future physician, or did it teach you something about your own limits and values? Was the impact of each incident, again, different?   [Response]   1. Looking back at such challenging moments and given the benefit of experience and hindsight, do you feel that your reactions to these events have changed as you’ve progressed through your training?   [Response] |
| **PIF and Internal Compass Formation** | 1. I am interested to know whether and how these experiences impacted your professional identity. Did these experiences influence the kind of doctor you see yourself becoming? Was the impact of each experience different (i.e., would you say one experience was. More significant than the other in terms of shaping your professional identity)? (Tease out the development of values/ beliefs/ approaches)   [Response]   1. Have these experiences ever make you question your career choice or path in medicine? (Or conversely, did overcoming these strengthen your commitment to your career?) Were there any other specific moments in your training when you felt your professional identity truly mature or evolve significantly?   Note:   - To tease out the impact of EACH experience – one experience might have strengthened interviewee’s resolve, the other might have weakened it - Interviewer to explore the idiosyncratic reflections by the interviewee   [Response]   1. As we’ve covered how these experiences might have shaped your professional identity, I’d like to ask if there’s anyONE in particular who has likewise shaped your professional identity and approach to challenges? (e.g., seniors, mentors, role models, whom you find yourself emulating?)   [Response]   1. Thinking back to when you first started medical training – how did the reality of these difficult experiences compare with your initial expectations and ideals? Were they challenged, or perhaps reinforced?   [Response] |
| **Impact of Experiences on Personhood using RToP** | Thank you for sharing about these cases and exploring their different aspects and emotional impact. I’d like to synthesise the impact that these cases have had on different facets of your personhood. Throughout this discussion, you may choose to think about your experiences in general, or you can delve deeper into specific instances if you’d like – both are more than welcome. You may also wish to think about yourself as a “whole person”, not just yourself as a medical student/ your professional side. We will structure this discussion using a framework that has 4 domains, like I mentioned at the start. The first domain is the innate domain, which refer to your core attributes, such as existential, spiritual, or religious beliefs or values.   1. Innate:  - How have your experiences in medical school/ training influenced the core beliefs and principles/ values that are important to who you are as a person, such as your existential or spiritual beliefs?   [Response]  Thank you, I would now like to explore how your experiences have influenced you as an individual. This is the second domain, for which I would like you to reflect on yourself as an autonomous being with the conscious abilities to think, talk and act autonomously.   1. Individual:  - How have your experiences influenced your sense of personal agency or autonomy as a person?   [Response]   - Do you feel that going through these tough experiences has changes your emotional reactions and emotional range? For example, do you feel you have become more guarded, more empathetic, more sensitive?   [Response]   - Do you feel that your self-awareness has increased as a result of these experiences? For example, do you feel you better understand your limits, triggers, and coping styles?   [Response]  Thank you, I would now like to explore how your experiences have impacted you in the next domain, which is the relational domain. This domain pertains to your relationships with those close to you.   1. Relational:  - How has your medical training influenced your relationships with the people close to you, such as with family and friends?   [Response]   - Have these relationships changed as a result of your experiences? For example, certain doctors’/ students’ experiences have strained their relationships because perhaps they bring the stress of work back home. Or conversely, some say that seeking emotional support from loved ones has strengthened their relationships. Do any of these resonate?   [Response]   - Have you gained any insights into the importance of personal relationships as a result of experiencing the stressful learning experiences?   [Response]  Thank you for sharing, now for the last domain, I would like to explore how your experiences have shaped your ties with society   1. Societal:  - In what ways have societal expectations or professional expectations within the medical community played a part in the way your experiences have unfolded? For example, you might feel pressure to continue working even when burnt out? Has there ever been a tension between societal or institutional expectations and your own personal values?   [Response]   - Has there ever been a tension between societal or institutional expectations and your own personal values? How did you manage this conflict?   [Response]   - How do you see your role as a doctor in society now, after going through these experiences? For example, you might feel a strengthened sense of duty and identity, or you may feel bogged down by the weight of public expectations?   [Response] |
| **Coping Mechanisms and Available Support** | 1. Given all the reflections we’ve just discussed, I’d like you to ponder about how you manage day-to-day stressors at work – do you have any strategies or routines to ensure you “unwind” properly? For example, routine exercise, routine meals with family? Any rituals to de-stress from work?   [Response]   1. Do you think your institution provides enough support and do you feel they are accessible and effective? If possible, ask to give positive/ negative examples of themselves or others they know using these supports   [Response]   1. Do you think the institution’s culture encourages medical students to seek help and prioritise well-being, or is there an unspoken expectation to “toughen it out”? And would you say that your attitudes and actions taken toward help-seeking has been shaped by those of your institution’s culture?   [Response]   1. What changes or additional supports do you think medical students would benefit from that schools could provide to foster healthy emotional coping and identity development? [E.g., mentorship programmes, forums, resilience curriculum]   [Response] |
| **Reflections and Looking Forward** | 1. We’ve covered a lot on your experiences and the learning points you’ve taken away from them. How do you anticipate that the lessons and experiences we’ve covered – and the ways you have learned to cope with them – will carry forward into your future practice and shape the kind of doctor you will be in, say, 5 or 10 years from now?   [Response]   1. Do you feel prepared for the emotionally challenging aspects of your future in medicine?   [Response]   1. Is there anything we haven’t talked about that you feel is important to mention regarding the emotional costs of learning, your identity formation, or your personal well-being? Or, anything you expected or wanted to discuss but we didn’t?   [Response]  Thank you for taking the time to do this interview today. Your perspectives help the medical education community understand and support the human side of healthcare training, which is very important not just for your well-being but for contributing to a cultural shift in how we view future healthcare professionals like you – as humans with limits, personhoods, stories, and experiences. Do you have any questions for me? |
